# Design and experimental evaluation of an orderly harvesting device for hydroponic leafy vegetables

**DOI:** 10.3389/fpls.2026.1909307

**Published:** 2026-08-07

**Authors:** Ruifeng Yu, Xianlong Wang, Jingwen Zhao, Yijing Zhang, Yidong Ma

**Affiliations:** 1 School of Intelligent Manufacturing Equipment, Henan Forestry Vocational College, Luoyang, China; 2 College of Agricultural Equipment Engineering, Henan University of Science and Technology, Luoyang, China

**Keywords:** clamping conveying, differential steering, hydroponic leafy vegetables, low-damage harvesting, orderly harvesting, root flicking

## Abstract

Hydroponic leafy vegetables are well suited to standardized cultivation, but mechanical harvesting remains constrained by root entanglement, unstable clamping and disordered plant posture. To address these problems, an orderly harvesting device integrating rotating root flicking, clamping conveying and differential steering was designed and evaluated using hydroponic spinach and Shanghai bok choy. The device used counter-rotating nylon brushes for non-cutting root treatment, a twisted double-belt clamping conveyor for low-damage conveying and a differential belt steering unit for posture adjustment. Multi-level single-factor tests were conducted for brush speed, clamping-conveying twist angle and upper steering-belt speed. The root removal rate increased from 65.7% to 79.2% as brush speed increased from 600 to 1000 r/min, while the damage rate increased from 1.5% to 4.1%; 800 r/min was selected as a balanced condition, giving a root removal rate of 77.4% and a damage rate of 2.5%. Under a twist angle of 60^◦^, a clamping gap of 24 mm, a conveying height difference of 200 mm and an upper steering-belt speed of 325 mm/s, spinach achieved a clamping-conveying success rate of 93.5%, a damage rate of 3.0% and a twist-angle MAE of 1.22^◦^, while Shanghai bok choy achieved 89.0%, 4.0% and 1.94^◦^, respectively. In differential steering, spinach achieved a steering success rate of 93.0%, a damage rate of 4.5% and an inclination-angle MAE of 1.15^◦^, while Shanghai bok choy achieved 94.5%, 3.5% and 0.98^◦^, respectively. The results indicate that the proposed device can complete coordinated root flicking, low-damage clamping conveying and posture steering for hydroponic leafy vegetables, providing a technical reference for orderly mechanical harvesting in controlled-environment production.

## 1 Introduction

Hydroponic and controlled-environment production systems are increasingly used for leafy vegetables because they support standardized crop spacing, short production cycles, predictable quality and efficient water and nutrient management (Nikolov et al., 2023; Ampim et al., 2022; Gumisiriza et al., 2022; Dutta et al., 2023; Barbosa et al., 2015). In vertical farming and plant-factory systems, these advantages are further amplified by environmental control and multilayer cultivation, although energy use, profitability and the need for automation remain major constraints for large-scale deployment (van Delden et al., 2021; Benke and Tomkins, 2017; Al-Kodmany, 2018; Kozai et al., 2019; Bunge et al., 2022; Baumont de Oliveira et al., 2022; Beacham et al., 2019). For leafy vegetables, mechanized harvesting is therefore not only a labor-saving measure but also an enabling technology for industrialized hydroponic production.

Compared with field-grown crops, hydroponic leafy vegetables are usually cultivated in trays, pipelines or planting holes with relatively uniform row and plant spacing. These agronomic features provide favorable boundary conditions for mechanized operation. However, the edible leaves and tender stems are highly susceptible to compression, friction and impact damage. Recent studies have explored aquatic-vegetable harvesters, hydroponic leafy-vegetable gripping mechanisms, machine-vision harvesting devices and automatic hydroponic lettuce systems (Guan et al., 2025; Liu et al., 2023; Khan et al., 2024; Ma et al., 2020; Praveen et al., 2025). A closely related MDPI study also demonstrated that root flicking, posture adjustment and differential steering can improve ordered harvesting of hydroponic leafy vegetables (Ma et al., 2026). Nevertheless, stable low-damage harvesting across different leafy-vegetable morphologies remains difficult because crop posture, clamping contact area and root distribution vary among species.

Root handling is a key step in hydroponic harvesting. The roots of hydroponic vegetables are long, soft and fibrous, and they can easily wrap around conveyors or neighboring plants. Physical and mechanical measurements of hydroponic lettuce and other leafy vegetables have shown that plant organs differ substantially in pulling force, cutting force, moisture content and viscoelastic response (Wang et al., 2021; Jin et al., 2024a). For hydroponic Chinese kale, finite-element calibration has been used to model stem cutting, while response-surface optimization has been applied to root-cutting tools for hydroponic lettuce (Xia et al., 2024; Gao et al., 2024). These studies indicate that harvesting mechanisms should be designed according to the mechanical properties of the crop rather than treated as rigid-body handling problems. In addition, operational parameters and water quality in closed hydroponic systems can affect root development and handling behavior (Hosseinzadeh et al., 2017).

Conventional vegetable root-removal mechanisms commonly use reciprocating cutters, disk cutters, rotary cutters or shoveling/cutting components. Such mechanisms have been reported for Chinese cabbage, garlic, chrysanthemum, mushrooms, corn roots and spinach root systems (Yu et al., 2025, 2023; Lu et al., 2025; Xie et al., 2025; Feng et al., 2025; Zhang et al., 2023; Zou et al., 2023). Although cutting can separate roots from stems, it may introduce compression, abrasion and posture disturbance, especially in leafy vegetables whose edible tissues are exposed during conveying. Brush-type or rubbing interactions provide an alternative non-cutting route for root-fiber removal. Prior studies on brush-root interactions and head-forming vegetable root-cutting devices have provided useful mechanical references (Sabiev et al., 2021; Li et al., 2025), but the integration of brush-based root flicking with subsequent clamping and posture steering for hydroponic leafy vegetables still requires further experimental validation.

After root treatment, the harvested plant must be conveyed with a stable posture. Orderly harvesting systems for leafy greens typically use clamping belts, flexible chains, compliant clamping mechanisms or auxiliary guiding devices. Flexible clamping and conveying devices have been developed to reduce plugging, mechanical clamping damage and transportation loss (Jin et al., 2024b). Rheological and viscoelastic models of spinach have also been used to design variable-stiffness compliant clamping mechanisms for low-damage harvesting (Zou et al., 2021). Domestic studies have proposed clamping-conveying devices for orderly leafy-vegetable harvesters and have analyzed spinach clamping based on rheological properties (Liu et al., 2025; Zou et al., 2019). However, plant posture during conveying remains stochastic. If the plant exits the clamping conveyor in a random orientation, subsequent sorting, packaging or collaborative robotic operations become unstable.

This study addresses the coupled problems of fibrous-root entanglement, low-damage clamping and posture consistency in hydroponic leafy-vegetable harvesting. An orderly harvesting device was developed by integrating a rotating-brush root-flicking mechanism, a clamping and twisting conveyor and a differential steering mechanism. Hydroponic spinach and Shanghai bok choy were selected as test crops because they differ in plant architecture and contact behavior. Root removal rate, harvesting success rate, visible damage rate and posture-angle MAE were used to evaluate the device. The objective was to determine whether coordinated root flicking, clamping conveying and differential steering could achieve stable, low-damage and orderly harvesting for hydroponic leafy vegetables.

## 2 Materials and methods

### 2.1 Agronomic characteristics of the test crops

**Figure 1:**
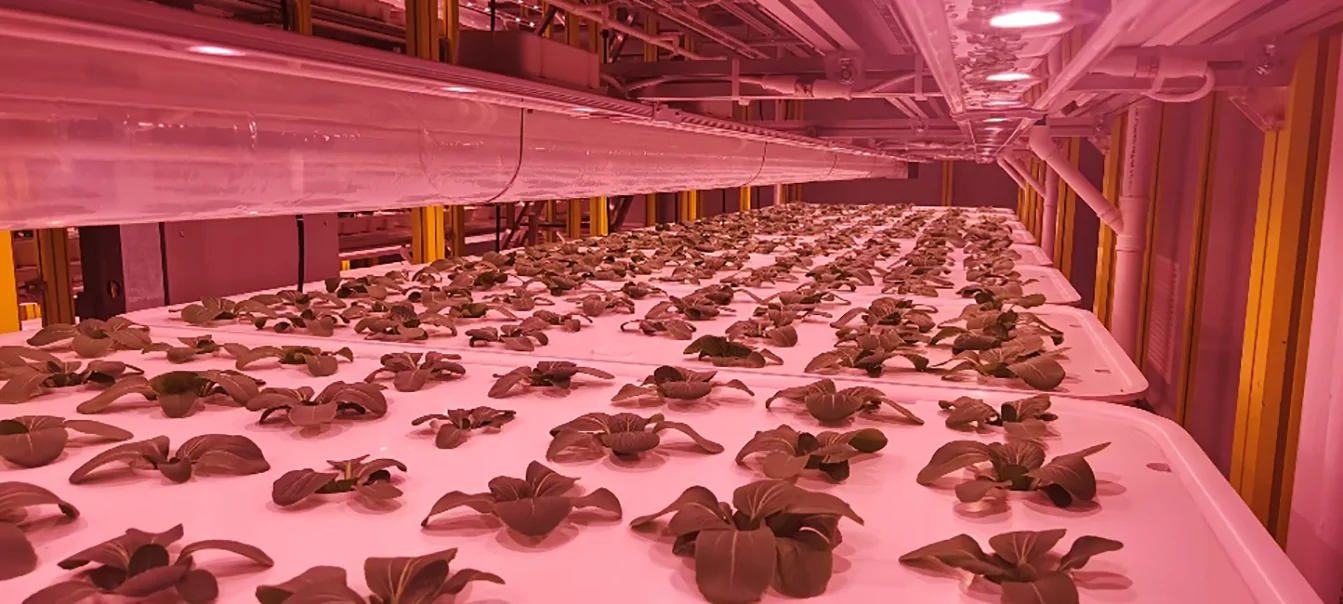
Agronomic characteristics of hydroponic leafy vegetables.

Hydroponic spinach and hydroponic Shanghai bok choy were used as experimental crops. As shown in **Figure 1**, both crops were cultivated in planting holes with single-row arrangement and uniform plant spacing. Plants at approximately 30 d after transplanting and with uniform growth status were selected. The two crops differed in canopy form, basal morphology and root distribution, and these differences were used to determine the adjustment range and operating parameters of the harvesting device. Spinach had a relatively open canopy, a higher plant height and lower single-plant fresh weight, while Shanghai bok choy had a shorter and more compact canopy, a larger basal contact region and greater single-plant fresh weight. The compact and heavier morphology of Shanghai bok choy increased the demand for stable clamping and posture control, whereas the larger leaf extension of spinach increased the risk of leaf interference during conveying.

**Figure 2:**
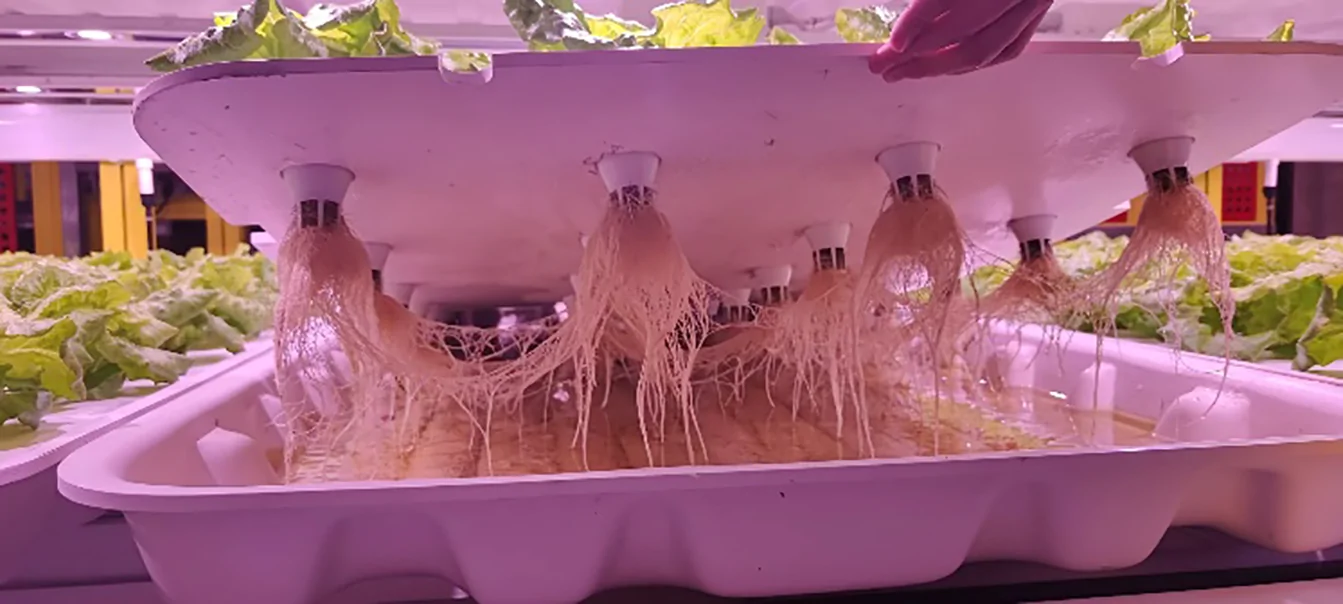
Root structure of hydroponic leafy vegetables.

The root system of hydroponic Shanghai bok choy differed markedly from soil-grown roots. As shown in **Figure 2**, the root system consisted mainly of numerous fibrous roots, without an obvious taproot. The roots were dense, long and soft, and therefore tended to entangle with adjacent plants and conveying components during harvesting. This root morphology was the main reason for selecting a non-cutting root-flicking mechanism before clamping and conveying.

The morphology measurements were used as geometric references for mechanism design rather than for inferential crop comparison. Therefore, two representative plants of each crop were measured after excluding abnormal individuals. For performance tests, the sample sizes were selected according to the role of each test. The kinematic tests based on high-speed video used 10 independent plants per factor level because each run required manual angle extraction and the purpose was to estimate angular deviation under controlled conditions. The root-flicking gradient test used 30 plants per brush-speed level because root length and root entanglement showed larger plant-to-plant variability. The 200-plant validation tests were used to estimate success rate and damage rate under continuous operation; for a success rate near 90%, this sample size gives an approximate standard error of about 2%, and for a damage rate of 3–5% it gives an approximate standard error of about 1–2%.

### 2.2 Overall structure and working principle of the harvesting device

**Figure 3:**
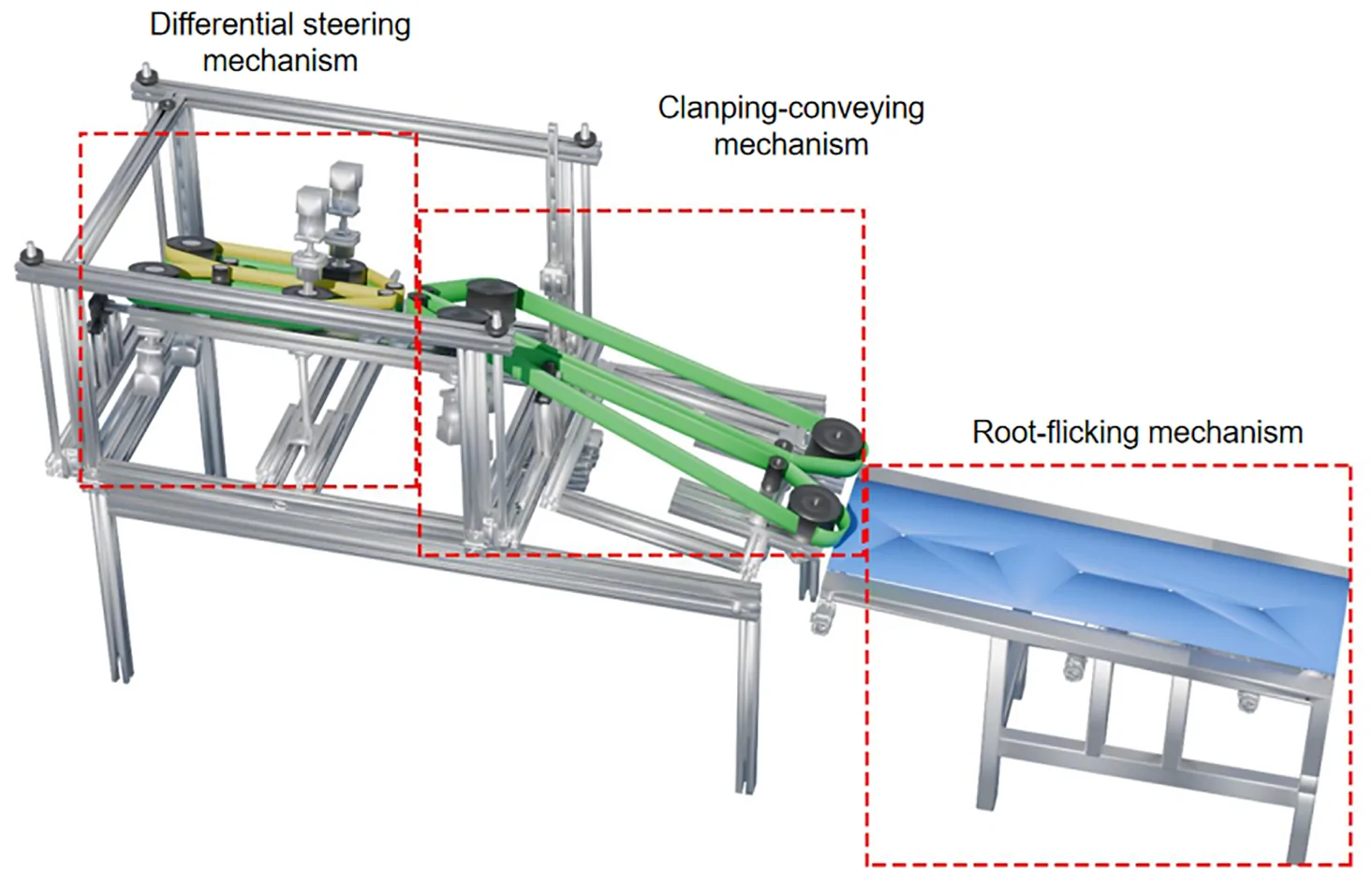
Overall structure of the hydroponic leafy-vegetable harvesting device.

The orderly harvesting device was designed using a modular layout (**Figure 3**). The overall harvesting plan consisted of crop inlet guidance, root treatment, clamping and conveying, differential posture steering and orderly output. During operation, the plants first enter the rotating root-flicking area, where excessive fibrous roots are shortened and disentangled. The plants are then guided into the double-belt clamping conveyor, which clamps the basal stem region and transports the plant while gradually twisting its posture. Finally, the plant enters the differential steering unit, where the speed difference between the upper and lower belts generates a backward inclination, so that the harvested plants leave the device with a more consistent orientation for subsequent collection or packaging.

The clamping conveyor consists of two synchronized polyurethane flat belts that stably clamp the plant stems and convey the plants continuously. The main operating parameters were a clamping gap of 24 mm, a twist angle of 60^◦^ and a height difference of 200 mm. The differential steering mechanism consists of two independently driven parallel belts. The lower belt speed was 250 mm/s, and the upper belt speed was adjustable from 250 to 400 mm/s. Under the selected working condition, the upper steering-belt speed was 325 mm/s, giving a speed difference of 75 mm/s. The root-flicking, clamping-conveying and differential steering modules were connected through the frame and adjustment mechanisms, allowing the inlet position, working height and steering posture to be matched to different crop sizes.

### 2.3 Design rationale for core mechanism selection

The core mechanisms were selected according to the morphological characteristics of hydroponic leafy vegetables and the requirement for continuous low-damage harvesting. A cutting-type root-removal device can rapidly separate roots, but the cutting edge must approach the root-neck region and may increase the risk of basal stem injury, leaf dragging and secondary contamination from cut root residues. By contrast, nylon bristle brushing applies distributed friction and impact to the flexible fibrous roots. This non-cutting contact is better suited to long and soft hydroponic roots because it reduces direct cutting of the edible stem region while shortening and disentangling the root mass before clamping.

For conveying, a double-belt clamping mechanism was selected instead of rigid grippers or roller pressing because polyurethane belts provide continuous contact and deformation compliance, which helps accommodate the crop-size differences shown in **Tables 1** and **2**. For posture correction, differential belt steering was selected instead of fixed guide plates, pneumatic pushing or intermittent robotic repositioning. Fixed guide plates are simple but can scrape leaves and have limited adaptability to crop size. Pneumatic pushing requires additional air supply and may cause unstable posture under variable canopy size. Intermittent robotic repositioning improves flexibility but reduces throughput and increases control complexity. Differential belt steering allows continuous posture adjustment through a controllable speed difference and can be integrated directly with the conveying path. The sample allocation for the experimental evaluation and the comparative analysis of the selected core mechanisms are presented in **Tables 3** and **4**, respectively. 

**Table 1:** Crop characteristics and their design implications.

Crop	Growth period	Plant type	Root characteristics	Design implication
Spinach	30 d	Relatively open canopy, larger leaf extension and lower single-plant mass	Fibrous root system with long, soft roots	Requires sufficient inlet width and compliant clamping to avoid leaf interference and stem indentation
Shanghai bok choy	30 d	Compact rosette canopy, larger basal contact region and higher single-plant mass	Dense fibrous roots, easy to entangle during harvesting	Requires stronger posture constraint, stable clamping and effective root cleaning before conveying

**Table 2:** Measured morphology of the two test crops.

Crop	*n*	Plant height (mm)	Canopy width (mm)	Basal width (mm)	Fresh weight (g)	Root length (mm)	Root fresh weight (g)
Spinach	2	259.0 ± 5.7	141.5 ± 3.5	27.0 ± 1.4	20.5 ± 0.6	166.5 ± 2.1	3.5 ± 0.3
Shanghai bok choy	2	180.0 ± 4.2	154.5 ± 2.1	40.0 ± 2.8	37.8 ± 0.6	148.5 ± 3.5	4.5 ± 0.1

Values are mean ± standard deviation.

**Table 3:** Sample allocation for the experimental evaluation.

Test item	Factor and levels	Crop	Sample size	Purpose
Morphology measurement	Representative crop dimensions	Spinach; Shanghai bok choy	2 plants per crop	Determine design envelope and adjustment range
Root-flicking gradient test	Brush speed: 600, 800 and 1000 r/min	Shanghai bok choy	30 plants per speed level	Compare root removal and root-flicking damage
Clampingconveying gradient test	Twist angle: 20^◦^, 40^◦^, 60^◦^ and 80^◦^	Spinach; Shanghai bok choy	10 plants per crop per level	Compare kinematic accuracy, success rate and damage rate
Differential steering gradient test	Upper belt speed: 275, 300, 325, 350 and 375 mm/s	Spinach; Shanghai bok choy	10 plants per crop per level	Compare posture steering stability and damage rate
Continuous validation	Selected operating condition	Spinach; Shanghai bok choy	200 plants per crop per process	Verify success rate and damage rate under continuous operation

**Table 4:** Comparative analysis of the selected core mechanisms.

Function	Selected mechanism	Alternative scheme	Main limitation of alternative	Reason for selection
Root cleaning	Counter-rotating nylon bristle brushes	Reciprocating or rotary cutting	Higher risk of basal stem cutting and leaf dragging near the rootneck region	Distributed flexible contact removes fibrous roots while reducing direct injury to edible tissues
Clamping and conveying	Twisted double polyurethane belts	Rigid grippers or roller pressing	Localized compression and poorer adaptation to crop- size variation	Continuous compliant clamping improves conveying stability and reduces indentation
Posture correction	Differential upper/lower belts	Fixed guide plates, pneumatic pushing or intermittent robotic repositioning	Scraping risk, unstable posture response or lower throughput	Adjustable speed difference enables continuous and controllable backward inclination

### 2.4 Root-flicking mechanism

**Figure 4:**
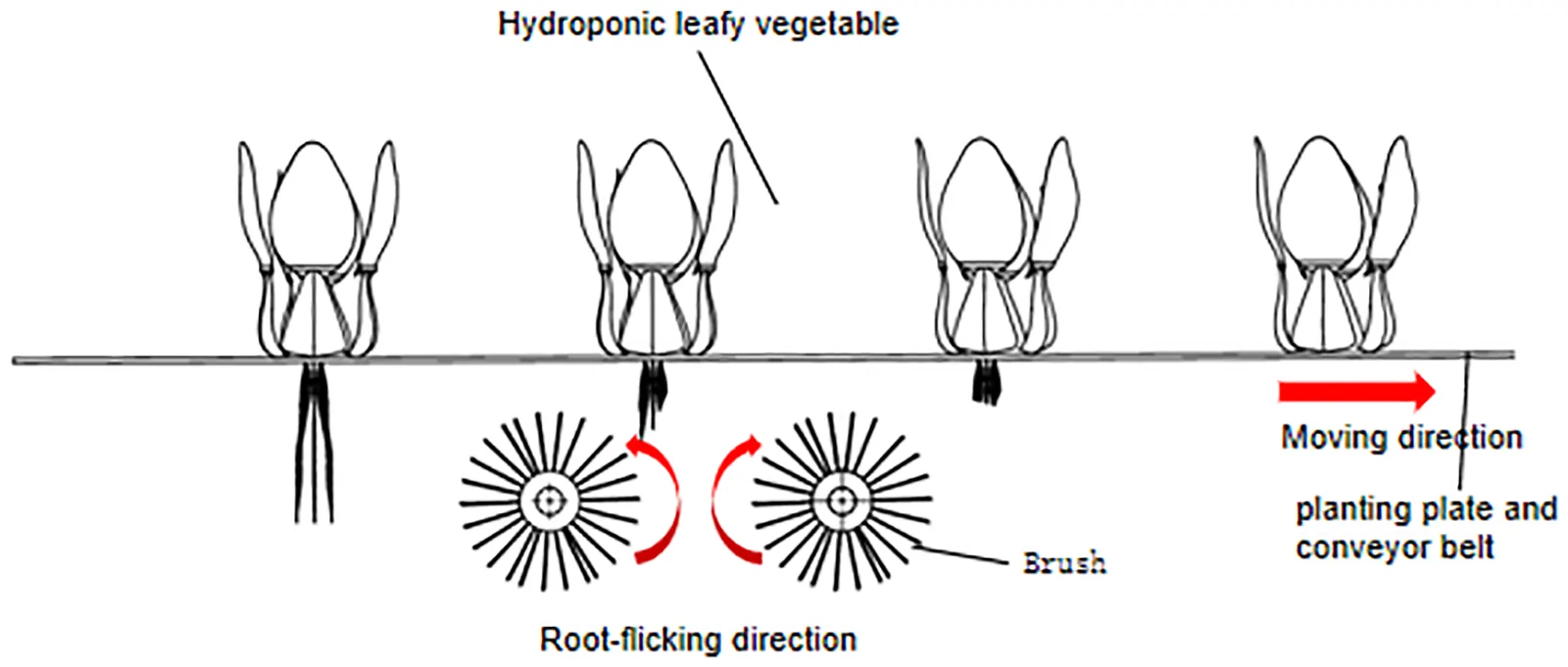
Design of the rotating root-flicking mechanism.

The rotating root-flicking mechanism is shown in **Figure 4**. It was installed at the front end of the conveyor inlet and consisted mainly of a conveyor belt, servo motors and two brush rollers. Nylon bristles were used to reduce mechanical injury during root cleaning. During operation, the plants were conveyed into the root-flicking area at 200 mm/s. The left brush rotated counterclockwise and the right brush rotated clockwise. The opposite rotation directions generated staggered friction and impact on the fibrous roots, which weakened the connection among long roots and shortened the root mass before clamping.

### 2.5 Clamping and conveying mechanism

**Figure 5:**
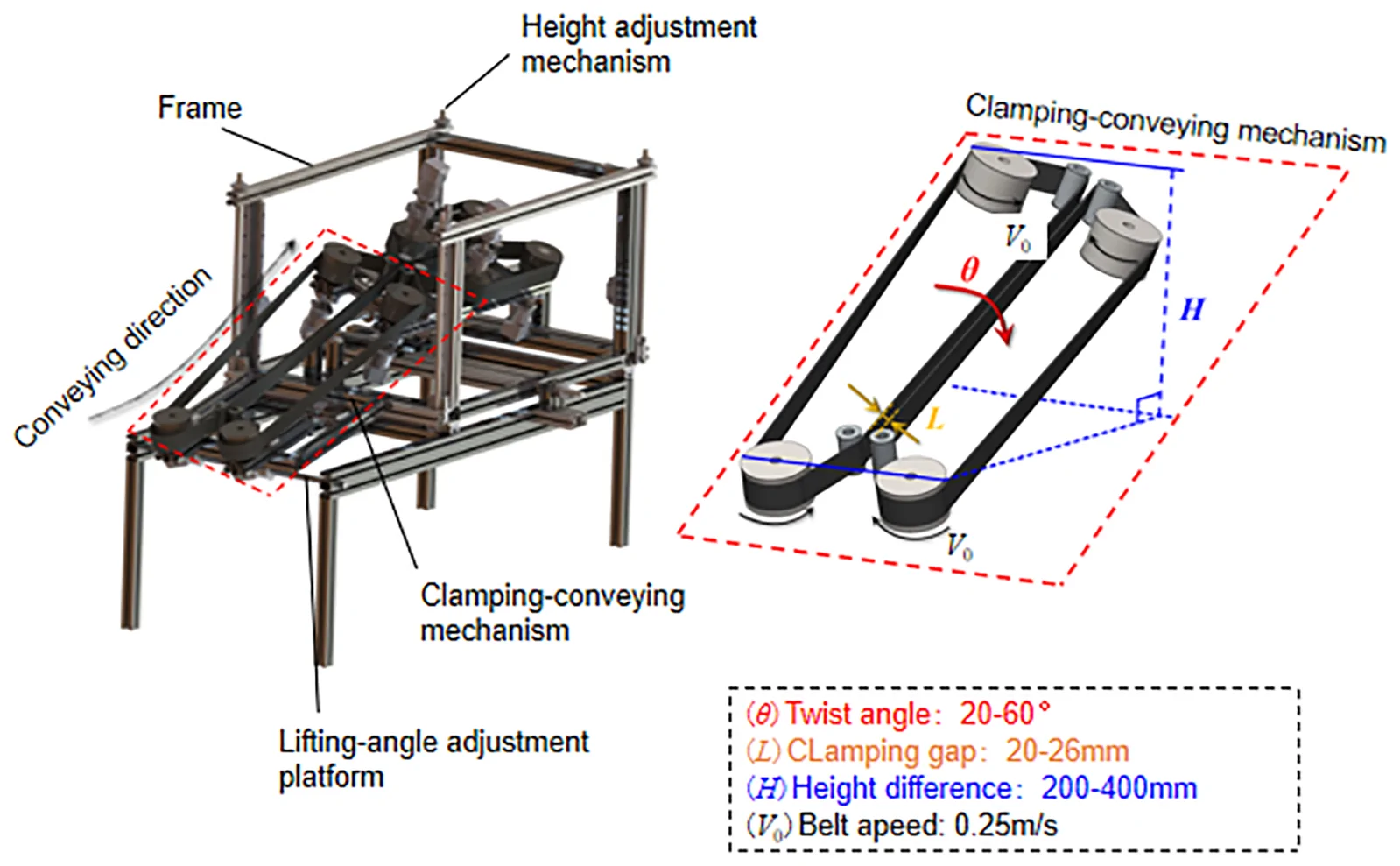
Clamping and conveying mechanism. The angle *θ* denotes the twist angle of the conveying path from inlet to outlet.

The clamping and conveying mechanism is shown in **Figure 5**. It mainly consisted of a driving wheel, driven wheel and polyurethane conveyor belt mounted on the frame. By arranging the conveying path with a controlled twist, the mechanism completed clamping, posture transition and continuous conveying. The key parameters included twist angle, clamping gap, conveying height difference and belt speed. The twist angle *θ* was defined as the rotation angle of the clamping-conveying path from the inlet plane to the outlet plane around the conveying direction. The clamping gap was the distance between the two belts at the basal stem contact region, and the height difference was the vertical distance between the inlet and outlet ends. These parameters were adjustable to match crop-size variation. The conveying belt speed was set to 250 mm/s to maintain stable conveying and reduce slippage caused by excessive speed.

### 2.6 Differential steering mechanism

**Figure 6:**
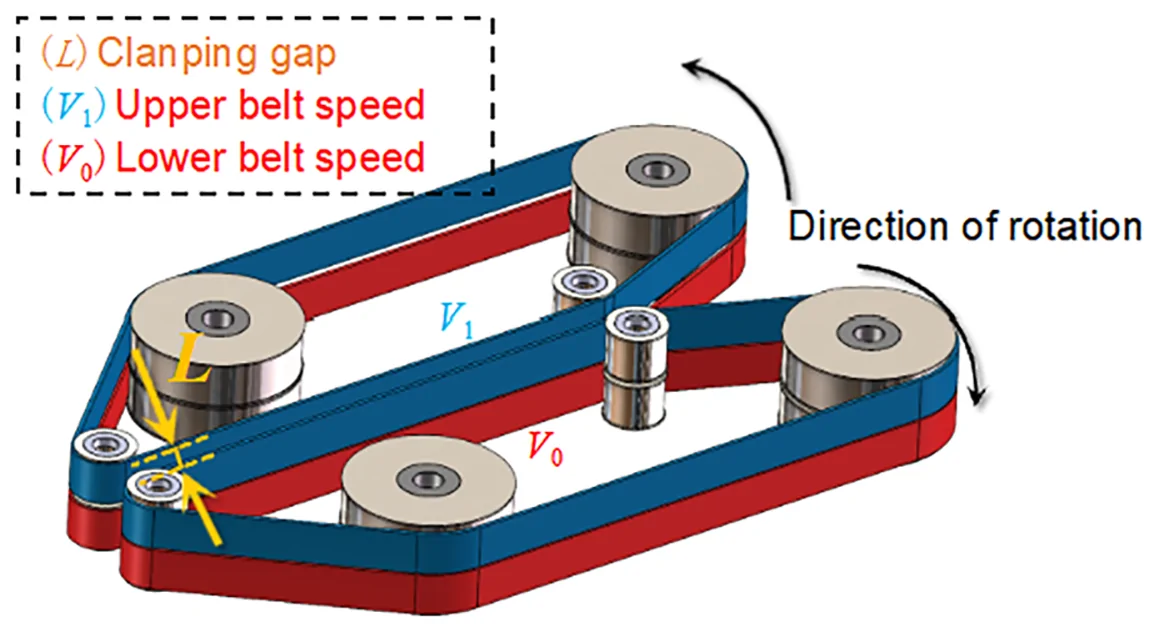
Differential steering mechanism for posture adjustment.

To satisfy the posture-consistency requirements of orderly harvesting and subsequent packaging, a differential steering mechanism was installed after the clamping conveyor (**Figure 6**). The mechanism consisted of upper and lower parallel belts driven independently. The lower belt supported and conveyed the lower part of the plant, whereas the upper belt pulled the upper part of the plant. When the upper belt speed was higher than the lower belt speed, a differential displacement was generated between the upper and lower parts of the plant, causing the plant to incline backward and leave the device with a more consistent posture.

The effective working length of the differential steering belts was 500 mm. In time *t*, the travel distance of the upper belt was calculated using Equation 1.

(1) $$L_{1}=V_{1}t,$$

where *L*_1_ is the travel distance driven by the upper belt, *V*_1_ is the upper steering-belt speed and *t* is the running time. The travel distance of the lower belt was calculated using Equation 2.

(2) $$L_{2}=V_{0}t,$$

where *L*_2_ is the travel distance driven by the lower belt and *V*_0_ is the lower conveying-belt speed. Since *V*_1_ *> V*_0_, the upper part of the plant moves farther than the lower part, resulting in backward inclination. In this study, *V*_0_ was fixed at 250 mm/s and *V*_1_ was adjustable from 250 to 400 mm/s.

### 2.7 Adjustment mechanisms

**Figure 7:**
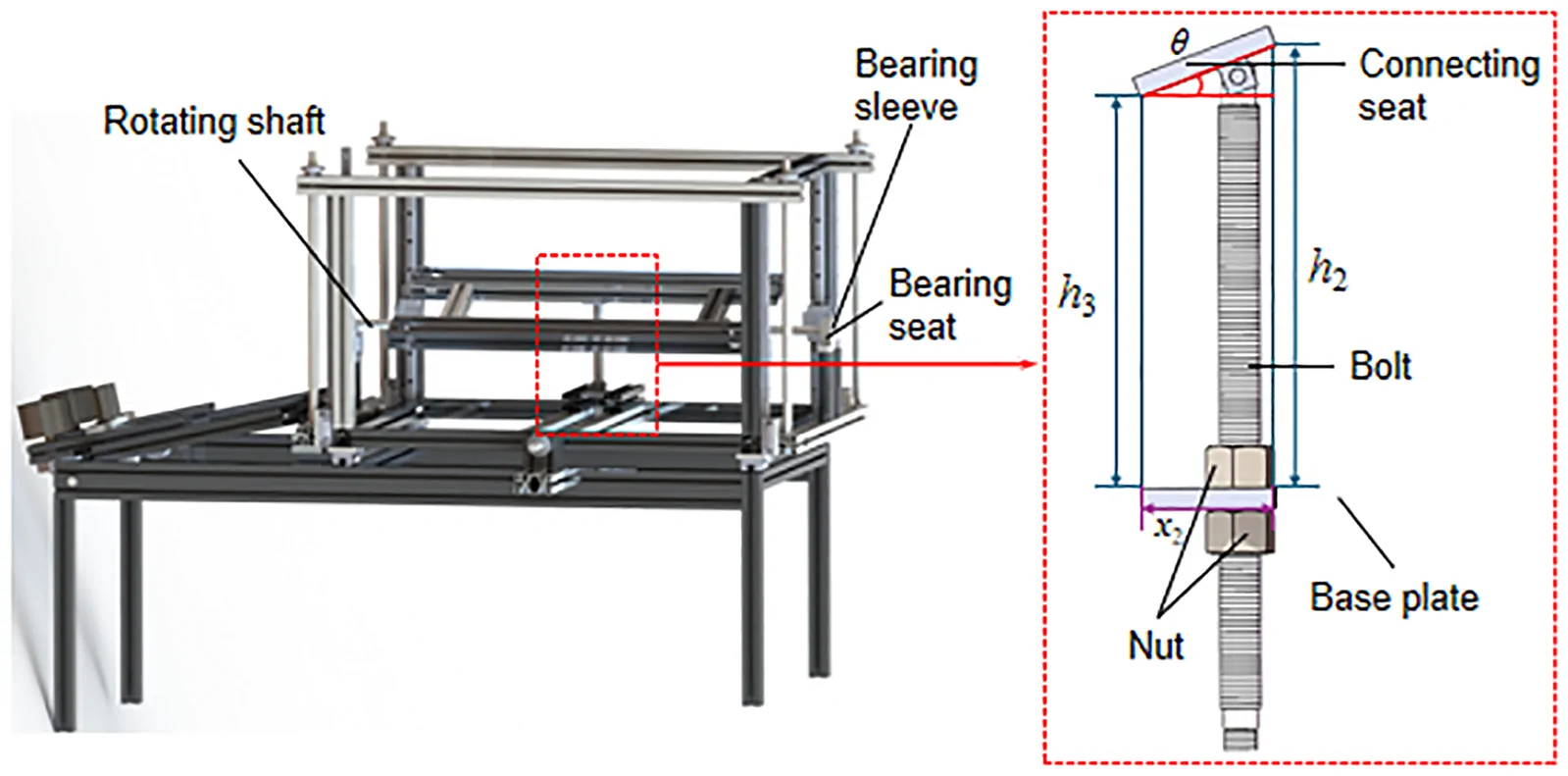
Twisting mechanism.

The twisting mechanism (**Figure 7**) consisted mainly of a support base, connecting seat, base plate, bolts, nuts, bearing sleeve and bearing seat. The support base fixed the differential steering mechanism, while the bearing sleeve and bearing seat allowed the frame to rotate around the shaft. By adjusting the bolt and nut positions, the frame and the differential steering unit rotated together, allowing the steering angle to be changed for different crop postures. This mechanism was used to set the twist angle before clamping-conveying and steering tests.

Let the base-plate length be *x*_2_, the distance from the left end of the upper base plate to the bottom of the connecting seat be *h*_3_ and the distance from the right end be *h*_2_. The twist angle was calculated using Equation 3.

(3) $$\theta =\text{arctan}\left(\frac{h_{2}-h_{3}}{x_{2}}\right).$$

The adjustable twist-angle range was 20–60^◦^, *h*_2_ ranged from 12.0 to 30.0 cm, *x*_2_ was 5.5 cm and *h*_3_ ranged from 10.0 to 28.0 cm. In the gradient tests, an 80^◦^ condition was also tested by increasing the relative installation angle of the clamping path to evaluate the effect of excessive twisting.

**Figure 8:**
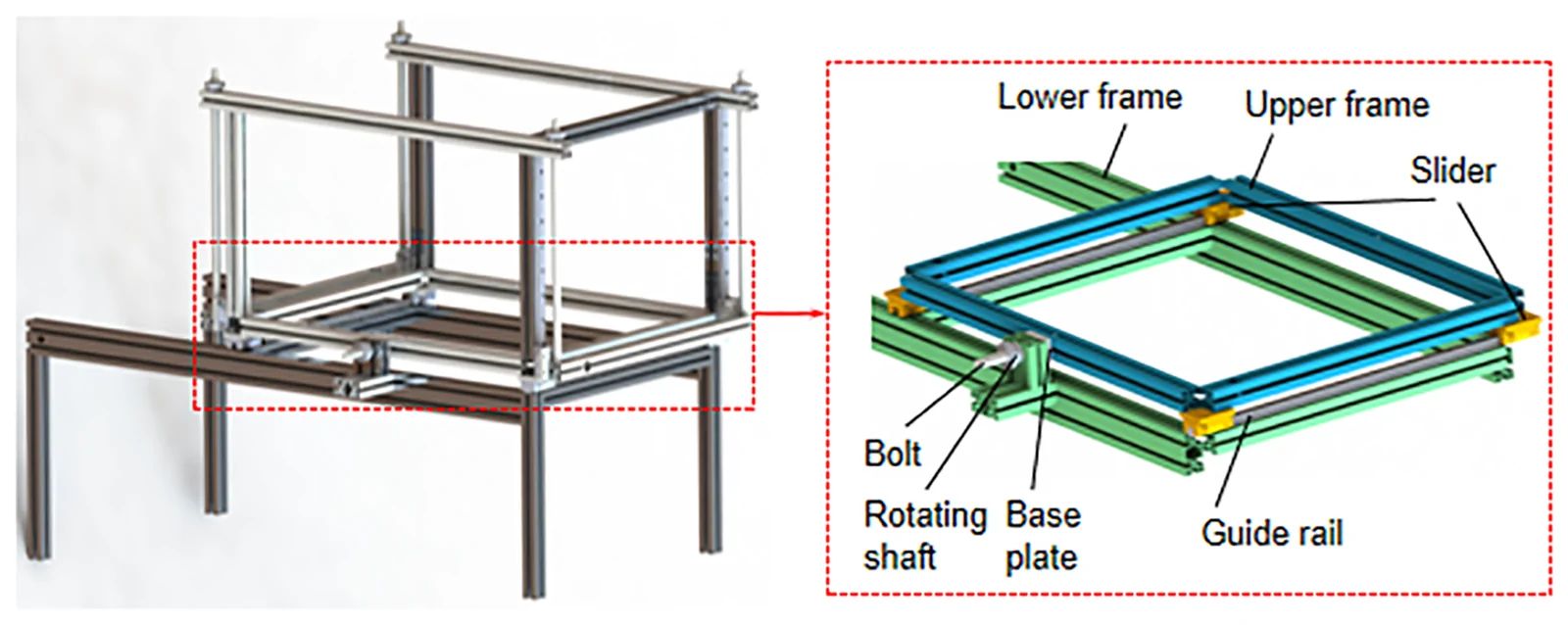
Lateral adjustment mechanism.

The lateral adjustment mechanism (**Figure 8**) consisted of bolts, a base plate, lateral adjustment frame, slider and guide rail. Rotating the adjustment bolt moved the base plate and frame along the guide rail, thereby changing the transverse position of the differential steering unit. This adjustment was used to align the steering belts with the outlet position of the clamping conveyor and to compensate for differences in crop canopy width and basal contact position. The lateral adjustment range was 0–10.0 cm.

**Figure 9:**
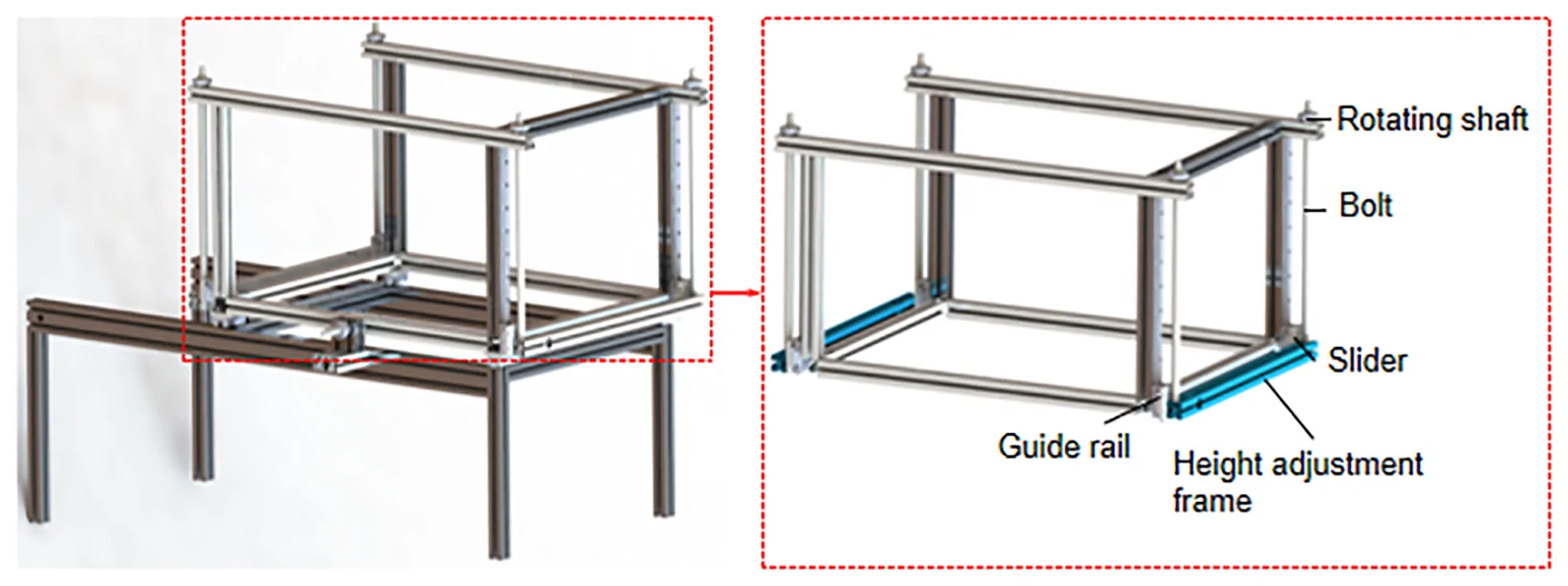
Height adjustment mechanism.

The height adjustment mechanism (**Figure 9**) consisted of an adjustment nut, bolt, slider, guide rail and height-adjustment frame. Rotating the adjustment nut converted screw motion into vertical movement of the frame, thereby changing the height of the differential steering unit. The purpose of this mechanism was to align the clamping and steering modules with the crop basal region and canopy height, avoiding excessive compression of leaves or loss of contact with the stem. The height adjustment range was 20.0–40.0 cm.

### 2.8 Root-flicking test

Root-flicking tests were conducted on a self-built hydroponic leafy-vegetable harvesting platform to evaluate root removal performance. Hydroponic Shanghai bok choy plants with similar growth status were selected, and their initial root lengths were measured before testing. The conveyor speed was fixed at 200 mm/s so that plants could pass through the root-flicking area continuously and stably. To evaluate the effect of brush speed, three brush-speed levels were tested: 600, 800 and 1000 r/min. Thirty plants were tested at each speed level. The root removal rate and root-flicking damage rate were used as evaluation indices. Root-flicking damage was counted when visible stem abrasion, root-neck tearing or leaf tearing caused by the brushing process was observed.

**Figure 10:**
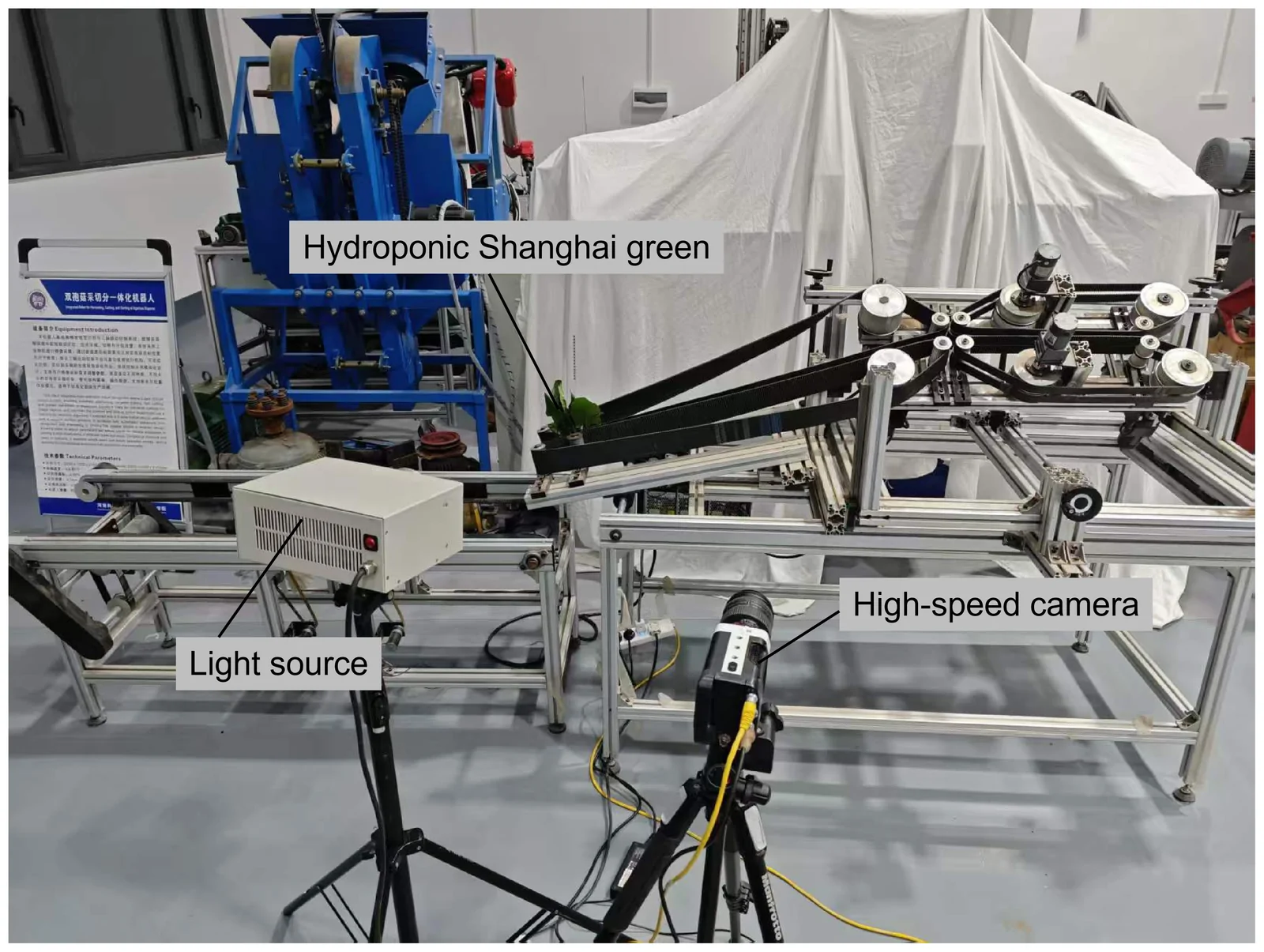
High-speed camera used to record the experimental process.

The root removal rate *η* was calculated using Equation 4.

(4) $$\eta =\frac{L_{\text{b}}-L_{\text{a}}}{L_{\text{b}}}\times 100\%,$$

where *L*_b_ is the root length before root flicking and *L*_a_ is the root length after root flicking. The root-flicking process was recorded using a high-speed camera (Miro LC111, 100 frames/s), as shown in **Figure 10**.

### 2.9 Clamping-conveying and differential steering tests

Clamping-conveying and differential steering tests were conducted to evaluate the adaptability of the device to the two crops. For clamping-conveying, the clamping gap was fixed at 24 mm and the height difference was fixed at 200 mm. Four twist-angle levels were tested: 20^◦^, 40^◦^, 60^◦^ and 80^◦^. At each angle, 10 spinach plants and 10 Shanghai bok choy plants were tested. The clamping-conveying success rate, damage rate and mean absolute error (MAE) between the theoretical and measured twist angles were used as evaluation indices. The MAE of the twist angle was calculated using Equation 5.

(5) $${\text{MAE}}_{\theta }=\frac{1}{n}\sum_{i=1}^{n}\left|\theta -\alpha_{i}\right|,$$

where *n* = 10, *θ* is the theoretical twist angle and *α_i_*is the measured twist angle in the *i*th test.

After the gradient comparison, an additional 200 spinach plants and 200 Shanghai bok choy plants were used for continuous clamping-conveying validation under the selected condition. The clamping-conveying success rate *S_x_* was calculated using Equation 6.

(6) $$S_{x}=\frac{S_{2}}{n}\times 100\%,$$

where *S*_2_ is the number of successful clamping-conveying operations and *n* is the number of test plants. The visible damage rate *D_x_* was calculated using Equation 7.

(7) $$D_{x}=\frac{D_{2}}{n}\times 100\%,$$

where *D*_2_ is the number of damaged plants.

For differential steering, the lower conveying-belt speed was fixed at 250 mm/s and the twist angle was fixed at 60^◦^. Five upper steering-belt speeds were tested: 275, 300, 325, 350 and 375 mm/s. At each speed, 10 spinach plants and 10 Shanghai bok choy plants were tested. The steering success rate, damage rate and MAE between the theoretical and measured inclination angles were used as evaluation indices. The MAE of the inclination angle was calculated using Equation 8.

(8) $${\text{MAE}}_{\beta }=\frac{1}{n}\sum_{i=1}^{n}\left|\beta -\gamma_{i}\right|,$$

where *n* = 10, *β* is the theoretical inclination angle and *γ_i_*is the measured inclination angle in the *i*th test. A further 200 spinach plants and 200 Shanghai bok choy plants were used for continuous differential steering validation under the selected condition. The steering success rate *S_y_* and damage rate *D_y_* were calculated using Equations 9 and 10, respectively.

(9) $$S_{y}=\frac{S_{3}}{n}\times 100\%,$$

(10) $$D_{y}=\frac{D_{3}}{n}\times 100\%,$$

where *S*_3_ is the number of successful steering operations and *D*_3_ is the number of damaged plants.

### 2.10 Damage grading and procedure-level recording

Damage was evaluated immediately after each working procedure, including root flicking, clampingconveying and differential steering. Visual damage was graded according to **Table 5**. Grade 0 indicated no observable damage. Grade 1 indicated slight damage that did not affect subsequent conveying or commercial appearance. Grade 2 indicated moderate damage with clear local tearing, indentation or abrasion. Grade 3 indicated severe damage, such as stem fracture, large leaf tearing or root-neck tearing that made the plant unsuitable for orderly output. In the calculation of damage rate, plants with Grade 1 or higher were counted as damaged. The procedure where the damage first appeared was recorded to distinguish root-flicking damage, clamping-conveying damage and steering damage.

**Table 5:** Damage grading criteria for hydroponic leafy vegetables.

Grade	Leaf damage	Stem or basal damage	Root or root-neck damage
0	No visible tearing, wrinkling or abrasion	No visible indentation or compression mark	No root-neck tearing or abnormal root pulling
1	Slight edge scratch or small local wrinkle	Slight indentation without tissue rupture	Slight root abrasion without root-neck tearing
2	Clear local tearing or abrasion, but plant remains conveyable	Clear indentation or local tissue damage	Partial root-neck tearing or obvious root abrasion
3	Large leaf tearing, folding or severe abrasion affecting commercial appearance	Stem fracture, severe compression or loss of clamping integrity	Severe root-neck tearing or plant failure during output

## 3 Results

### 3.1 Root-flicking test results

The root-flicking results are shown in **Table 6**. As brush speed increased from 600 to 1000 r/min, the root removal rate increased from 65.7% to 79.2%, whereas the root-flicking damage rate increased from 1.5% to 4.1%. This indicates that increasing brush speed enhanced the friction and impact applied to fibrous roots, but excessive speed also increased the risk of mechanical injury. At 800 r/min, the root removal rate reached 77.4%, which was close to that at 1000 r/min, while the damage rate was lower (2.5% versus 4.1%). Therefore, 800 r/min was selected as the balanced brush speed for subsequent tests.

**Table 6:** Root-flicking results under different brush speeds.

Brush speed (r/min)	Mean root length before flicking (mm)	Mean root length after flicking (mm)	Root removal rate (%)	Root-flicking damage rate (%)
600	149.8	51.4	65.7	1.5
800	150.2	33.9	77.4	2.5
1000	151.3	31.5	79.2	4.1

**Figure 11:**
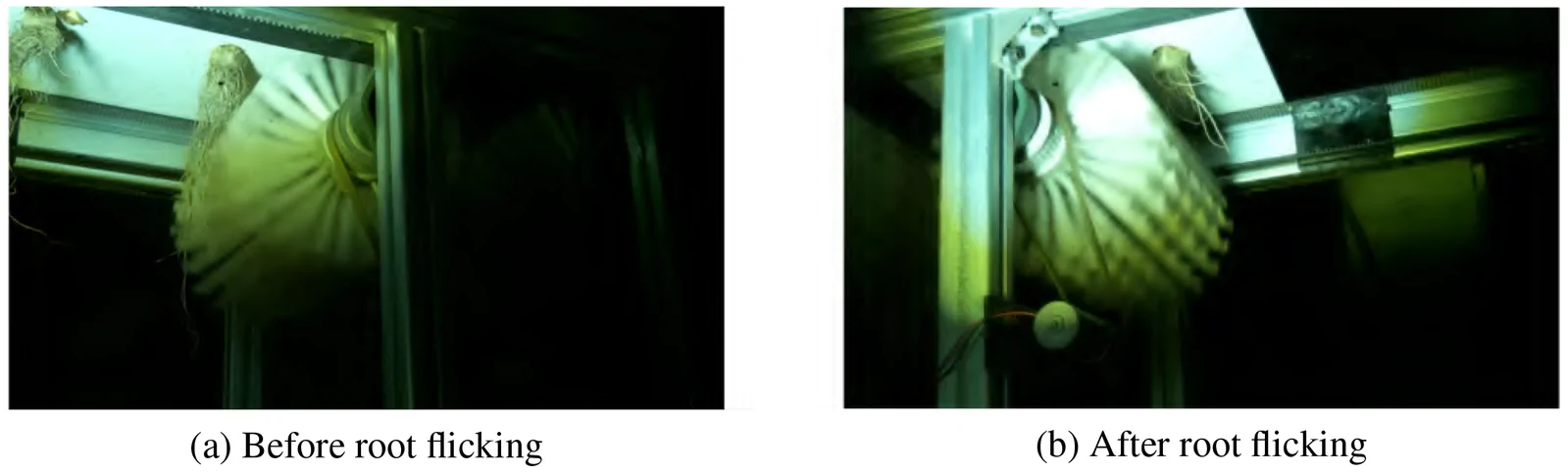
Comparison of roots before and after root flicking: **(a)** before root flicking; **(b)** after root flicking.

**Figure 11** shows the comparison of root condition before and after the root-flicking process. Before root flicking, the hydroponic Shanghai bok choy roots were long and dense. After rotating-brush treatment, only part of the thicker roots remained, and the long fibrous roots were effectively shortened.

Under the counter-rotating brush arrangement, the two brushes produced staggered contact with the roots. The fibrous root mass was subjected to continuous friction and alternating impact within a short time, which weakened root entanglement and improved root removal while avoiding direct cutting of the basal stem region.

### 3.2 Clamping-conveying performance

**Table 7** shows the clamping-conveying results of spinach under different twist angles. As the twist angle increased from 20^◦^ to 60^◦^, the success rate increased from 76.5% to 93.5%, and the twist-angle MAE decreased from 3.42^◦^ to 1.22^◦^. When the twist angle increased to 80^◦^, the success rate decreased to 91.0% and the damage rate increased to 5.5%. This indicates that a moderate twist angle improved posture transition and clamping stability, whereas excessive twisting increased stem torsion and damage risk. Therefore, 60^◦^ was selected as the preferred twist angle for spinach.

**Table 7:** Spinach clamping-conveying results under different twist angles.

Twist angle, *θ* (^◦^)	Success rate (%)	Damage rate (%)	Mean measured twist angle, *α* (^◦^)	MAE (^◦^)
20	76.5	1.2	17.8	3.42
40	86.0	2.0	38.5	2.18
60	93.5	3.0	58.7	1.22
80	91.0	5.5	76.3	2.56

**Figure 12:**
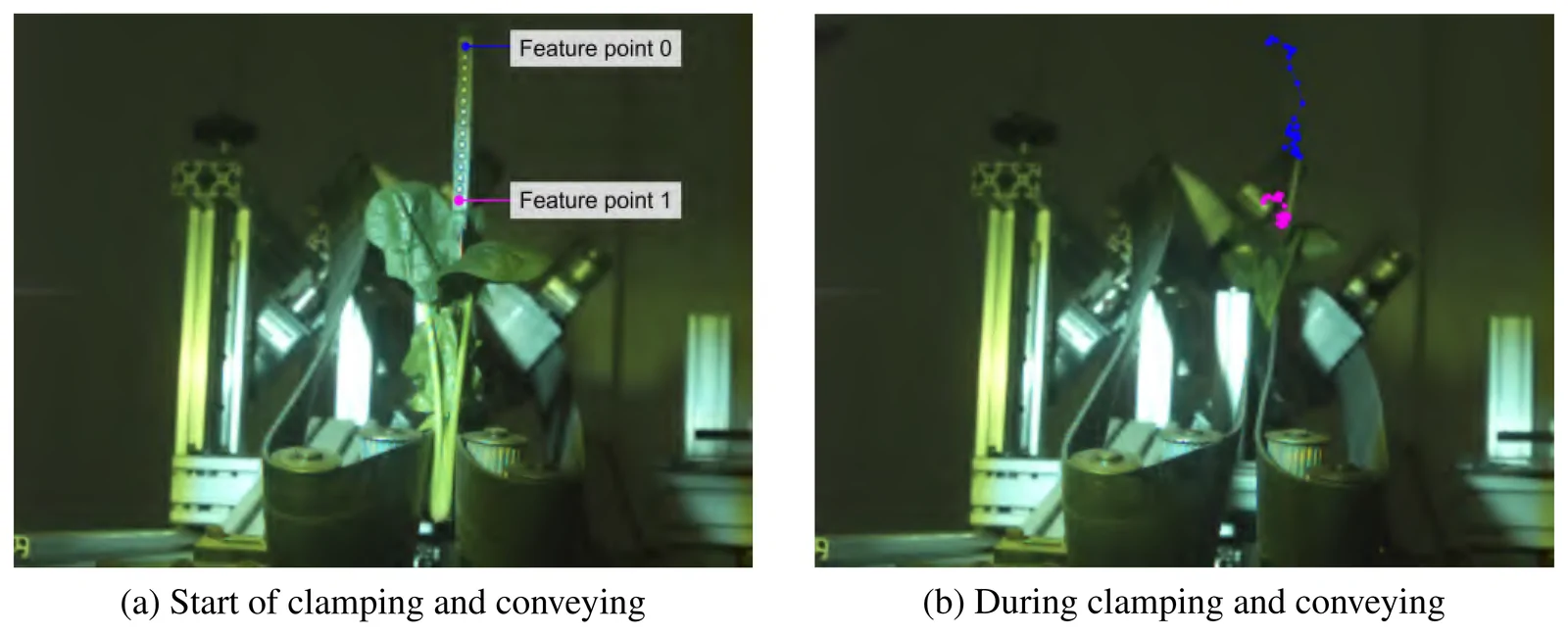
Motion trajectory of spinach during clamping and conveying: **(a)** start of clamping and conveying; **(b)** during clamping and conveying.

**Figure 13:**
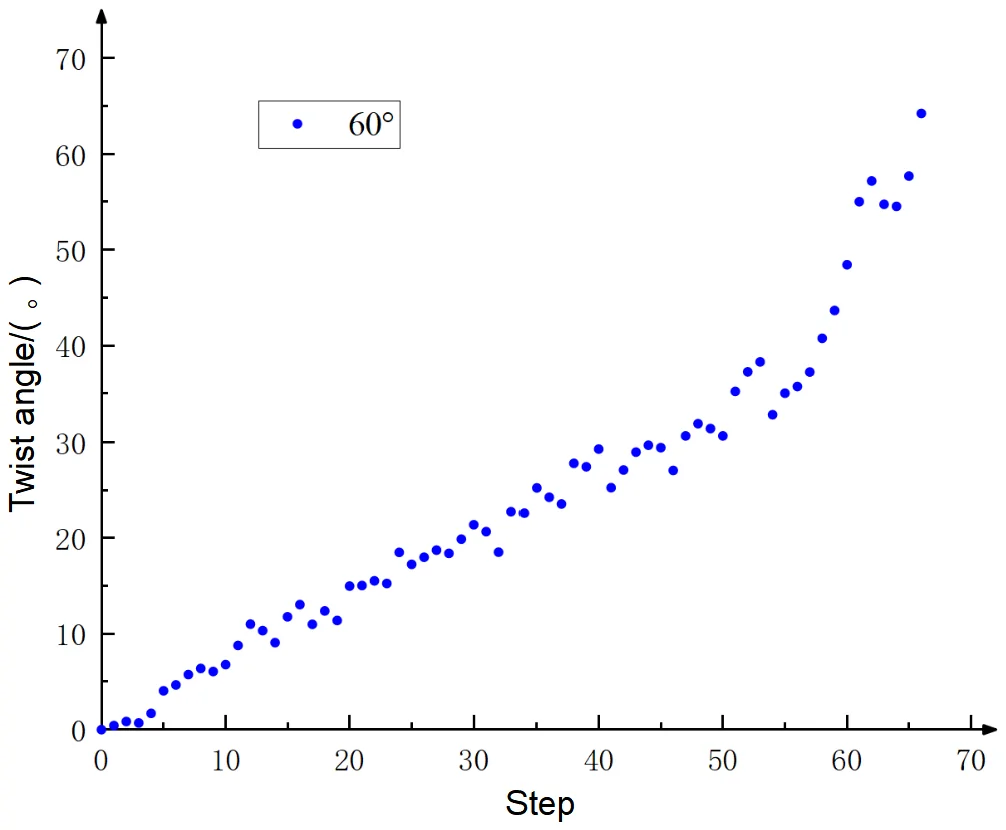
Example of twist-angle variation in spinach during clamping and conveying.

**Figure 12** shows the motion trajectory of spinach during clamping and conveying, and **Figure 13** presents an example of twist-angle variation during the process.

At the selected twist angle of 60^◦^, spinach moved continuously without obvious slippage or detachment. In the 200-plant continuous validation test, the clamping-conveying success rate reached 93.5% and the damage rate was 3.0%, showing that the clamping conveyor provided stable low-damage conveying for spinach.

**Table 8** shows the clamping-conveying results of Shanghai bok choy under different twist angles. Similar to spinach, the highest success rate (89.0%) and the lowest MAE (1.94^◦^) were obtained at 60^◦^. The success rate of Shanghai bok choy was slightly lower than that of spinach at each twist angle, and the damage rate increased to 6.0% at 80^◦^. This was attributed to the compact canopy and larger basal contact region of Shanghai bok choy, which made local contact forces more concentrated during twisting. Therefore, 60^◦^ was also selected as the preferred twist angle for Shanghai bok choy.

**Table 8:** Shanghai bok choy clamping-conveying results under different twist angles.

Twist angle, *θ* (^◦^)	Success rate (%)	Damage rate (%)	Mean measured twist angle, *α* (^◦^)	MAE (^◦^)
20	78.0	1.5	18.6	3.85
40	84.5	2.5	39.2	2.76
60	89.0	4.0	56.5	1.94
80	87.0	6.0	75.0	3.12

**Figure 14:**
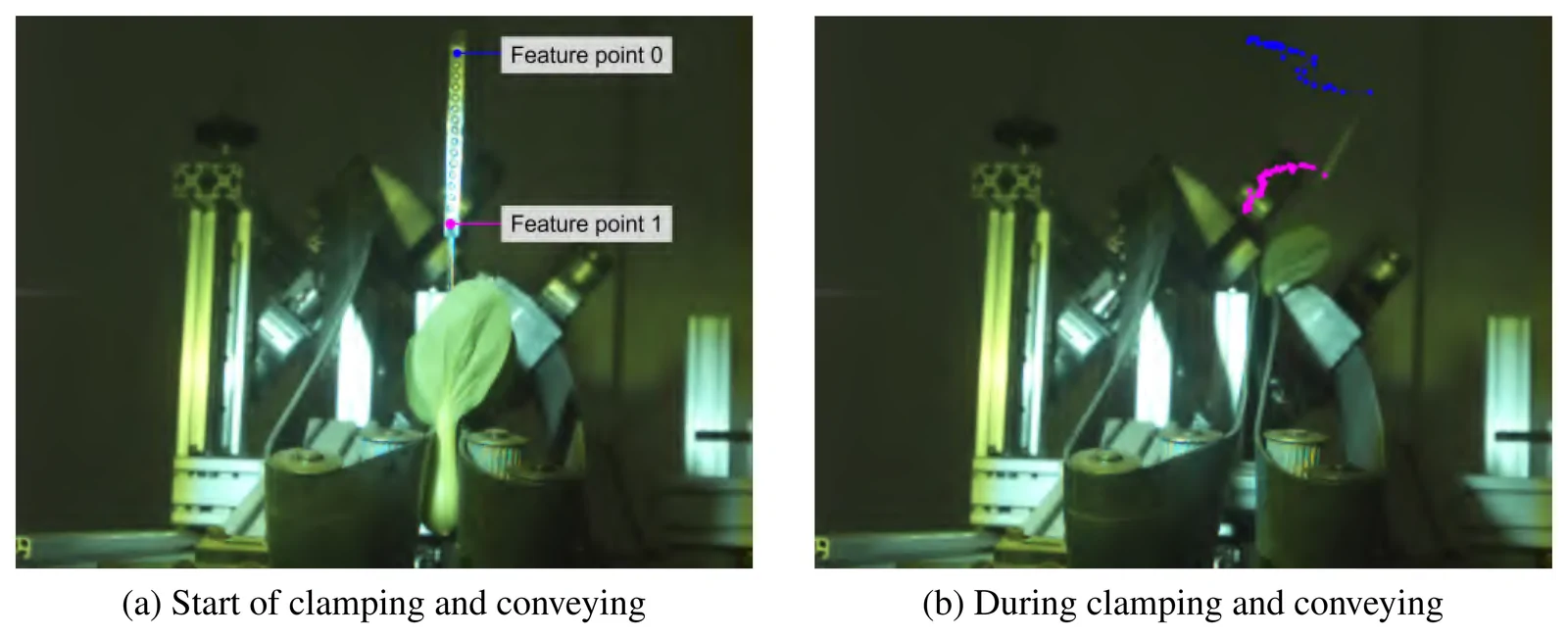
Motion trajectory of Shanghai bok choy during clamping and conveying: **(a)** start of clamping and conveying; **(b)** during clamping and conveying.

**Figure 15:**
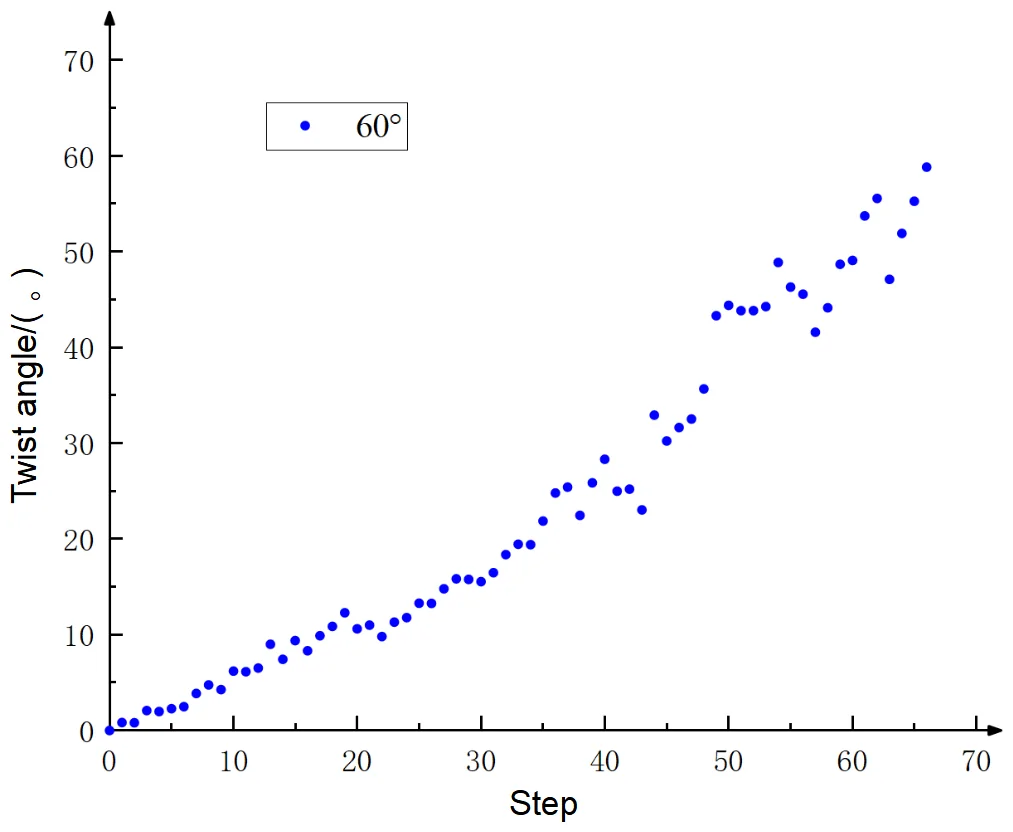
Example of twist-angle variation in Shanghai bok choy during clamping and conveying.

**Figure 14** shows the motion trajectory of Shanghai bok choy during clamping and conveying, and **Figure 15** presents the twist-angle variation.

At the selected twist angle of 60^◦^, Shanghai bok choy could be clamped and conveyed continuously, although the twist-angle trajectory was more dispersed than that of spinach. In the 200-plant continuous validation test, the success rate was 89.0% and the damage rate was 4.0%, indicating that the clamping conveyor maintained acceptable stability for the more compact crop type.

### 3.3 Differential steering performance

**Table 9** shows the differential steering results of spinach under different upper steering-belt speeds. When the upper belt speed increased from 275 to 325 mm/s, the steering success rate increased from 87.5% to 93.0%, and the inclination-angle MAE decreased from 2.85^◦^ to 1.15^◦^. When the belt speed exceeded 325 mm/s, the damage rate increased and the success rate decreased. This indicates that an appropriate speed difference generated sufficient steering torque, whereas excessive speed increased leaf pulling and posture instability. Therefore, 325 mm/s was selected as the preferred upper steering-belt speed for spinach.

**Table 9:** Spinach differential steering results under different upper steering-belt speeds.

Upper belt speed (mm/s)	Success rate (%)	Damage rate (%)	Mean measured inclination angle, *γ* (^◦^)	MAE (^◦^)
275	87.5	2.5	62.3	2.85
300	91.0	3.5	66.5	1.72
325	93.0	4.5	68.7	1.15
350	91.5	5.5	67.2	1.48
375	88.0	7.0	64.0	2.31

**Figure 16:**
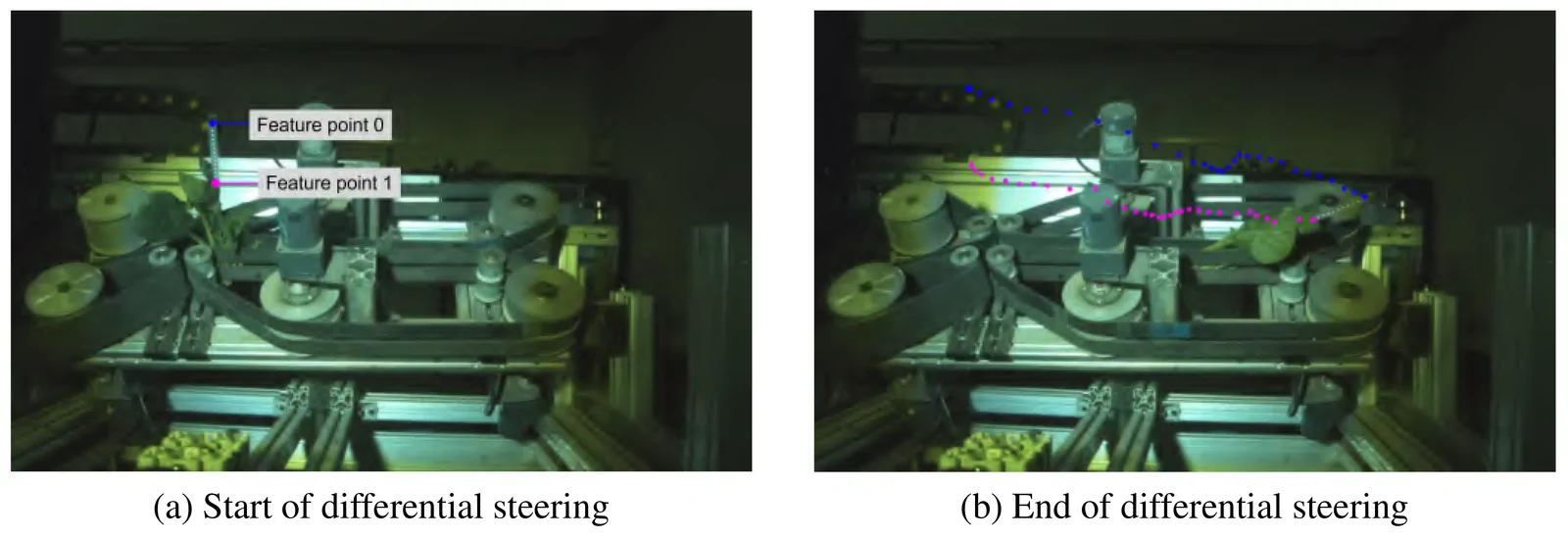
Motion trajectory of spinach during differential steering: **(a)** start of differential steering; **(b)** end of differential steering.

**Figure 17:**
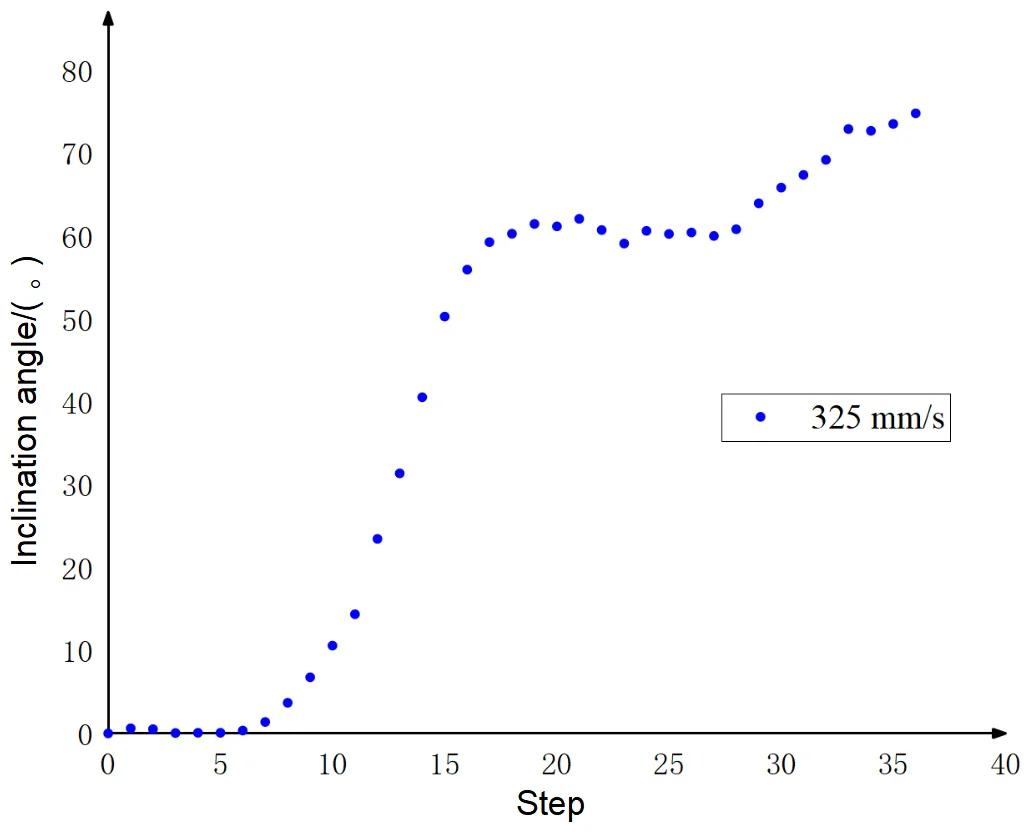
Example of inclination-angle variation in spinach during differential steering.

**Figure 16** shows the motion trajectory of spinach during differential steering, and **Figure 17** presents an example of inclination-angle variation.

At the selected upper belt speed of 325 mm/s, spinach maintained stable posture transition. In the 200-plant continuous validation test, the steering success rate was 93.0%, the damage rate was 4.5% and the inclination-angle MAE was 1.15^◦^.

**Table 10** shows the differential steering results of Shanghai bok choy under different upper steering-belt speeds. The highest success rate (94.5%) and the lowest MAE (0.98^◦^) were also obtained at 325 mm/s. Compared with spinach, Shanghai bok choy showed slightly higher steering success rates and lower MAE values, indicating that the compact crop type maintained more stable posture during differential steering.

**Table 10:** Shanghai bok choy differential steering results under different upper steering-belt speeds.

Upper belt speed (mm/s)	Success rate (%)	Damage rate (%)	Mean measured inclination angle, *γ* (^◦^)	MAE (^◦^)
275	89.0	2.0	64.7	2.43
300	92.5	2.5	68.1	1.56
325	94.5	3.5	69.6	0.98
350	93.0	5.0	68.4	1.32
375	89.5	6.5	65.2	1.98

**Figure 18:**
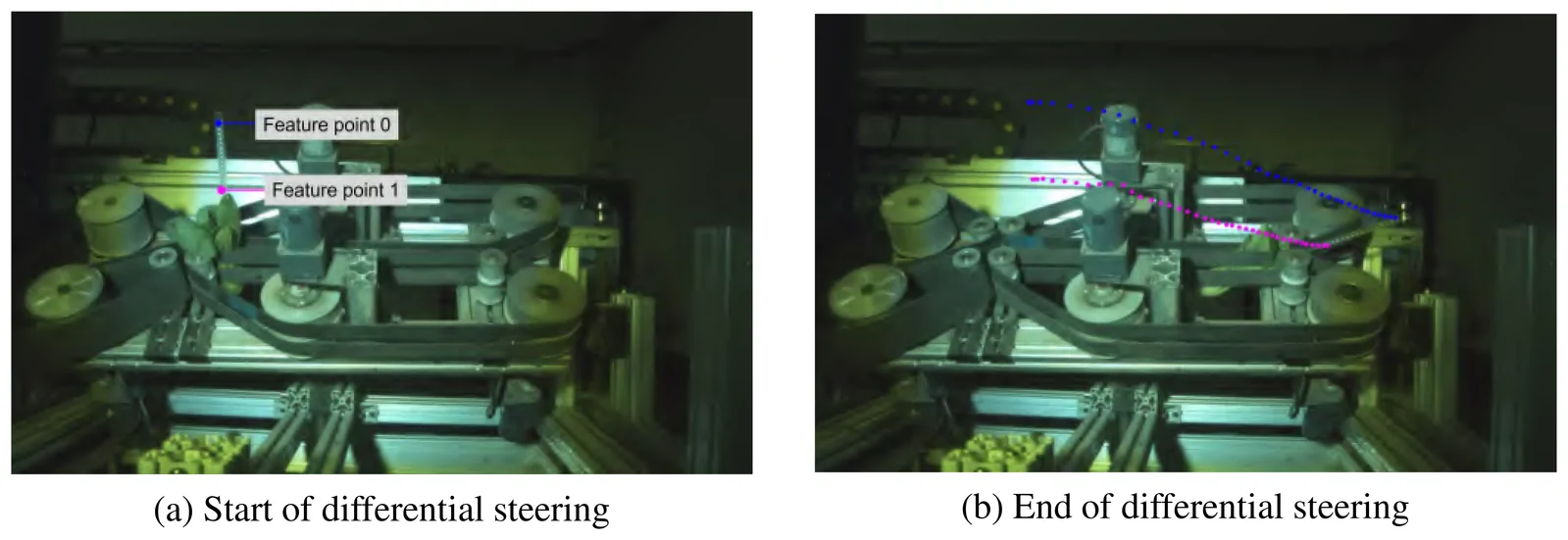
Motion trajectory of Shanghai bok choy during differential steering: **(a)** start of differential steering; **(b)** end of differential steering.

**Figure 19:**
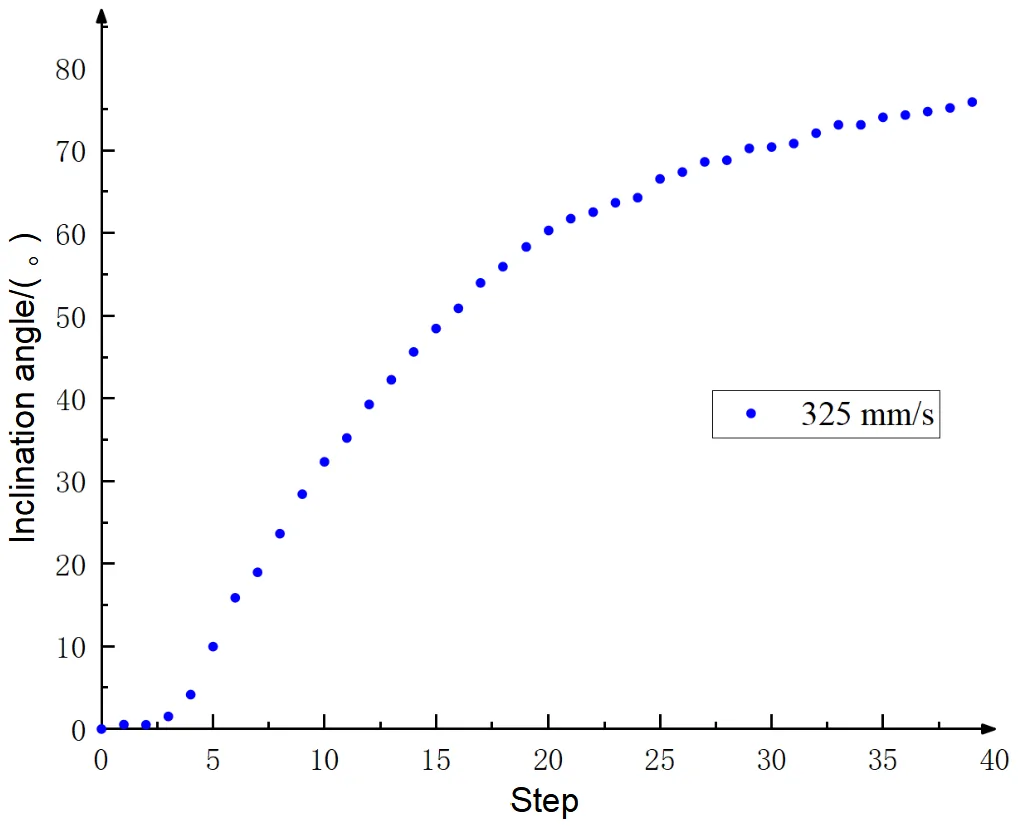
Example of inclination-angle variation in Shanghai bok choy during differential steering.At the selected upper belt speed of 325 mm/s, Shanghai bok choy achieved a steering success rate of 94.5%, a damage rate of 3.5% and an inclination-angle MAE of 0.98^◦^ in the 200-plant continuous validation test.

**Figure 18** shows the motion trajectory of Shanghai bok choy during differential steering, and **Figure 19** presents an example of inclination-angle variation. Shanghai bok choy maintained stable contact with the steering belts and gradually inclined toward the target posture.

### 3.4 Procedure-level damage summary

The procedure-level damage results under the selected working condition are summarized in **Table 11**. Root-flicking damage was mainly slight root abrasion or local root-neck tearing. Clamping-conveying damage was mainly slight stem indentation or local leaf abrasion caused by belt contact. Differential steering damage was mainly caused by leaf pulling or local compression during posture correction. The damage rate of each procedure remained below 5% under the selected working condition.

**Table 11:** Procedure-level damage rates under the selected working condition.

Procedure	Crop	Selected parameter	Damage rate (%)	Main observed damage type
Root flicking	Shanghai bok choy	800 r/min	2.5	Slight root abrasion and occasional root-neck tearing
Clampingconveying	Spinach	60^◦^ twist angle	3.0	Slight stem indentation or leaf-edge abrasion
Clampingconveying	Shanghai bok choy	60^◦^ twist angle	4.0	Local basal compression and leaf abrasion
Differential steering	Spinach	325 mm/s belt upper speed	4.5	Leaf pulling and local compression
Differential steering	Shanghai bok choy	325 mm/s belt upper speed	3.5	Local leaf abrasion and slight basal compression

## 4 Discussion

The results show that the coordinated root-flicking, clamping-conveying and differential steering design can satisfy the basic requirements of orderly harvesting for two hydroponic leafy vegetables. The root-flicking gradient test confirmed that brush speed directly affected the balance between root removal and damage. Although 1000 r/min produced the highest root removal rate, the improvement over 800 r/min was small and was accompanied by a higher damage rate. Therefore, the selected brush speed of 800 r/min represents a compromise between root-cleaning efficiency and low-damage operation.

The clamping-conveying results indicate that twist angle is a key factor influencing posture control. A small twist angle was insufficient to complete posture transition, leading to lower success rates and larger angular error. Excessive twisting increased stem deformation and damage risk. For both spinach and Shanghai bok choy, 60^◦^ provided the best overall balance. The difference between crops was related to morphology. Spinach had a more open canopy and lower mass, which facilitated clamping and posture transition. Shanghai bok choy had a compact canopy and larger basal contact region, which made the contact force more concentrated and slightly reduced clamping-conveying stability.

The differential steering tests show that upper belt speed affected the steering torque applied to the upper part of the plant. At 275 and 300 mm/s, the speed difference was insufficient for stable and accurate backward inclination. At 350 and 375 mm/s, excessive traction increased damage and reduced stability. Therefore, 325 mm/s was the preferred upper belt speed for both crops. Shanghai bok choy showed slightly better steering stability than spinach, probably because its compact canopy moved more as an integrated body during belt contact, whereas spinach leaves were more dispersed and more susceptible to local pulling.

The mechanism comparison supports the use of bristle root flicking and differential belt steering for hydroponic leafy vegetables. Bristle brushing is suitable for fibrous hydroponic roots because it acts on roots through flexible distributed contact rather than direct cutting. Differential belt steering is compatible with continuous conveying and avoids the intermittent action of robotic repositioning or the scraping risk of fixed guide plates. The adjustment mechanisms further improve adaptability by allowing transverse and height alignment between modules and crop morphology.

### 4.1 Limitations and future work

The prototype still has several limitations. First, the present tests were conducted under controlled laboratory feeding conditions, and the initial plant position was relatively regular. In practical production, plant posture, root moisture content and local entanglement may vary more widely, which may affect clamping entry and steering consistency. Second, the parameter tests used single-factor gradients. This design clarified the individual effects of brush speed, twist angle and upper belt speed, but it did not quantify interactions among brush speed, clamping gap, twist angle, height difference and belt speed. Third, only spinach and Shanghai bok choy were tested. Although the two crops represented open-canopy and compact-canopy leafy vegetables, additional varieties with different stem stiffness, canopy width and root morphology are needed to verify broader adaptability. Fourth, damage evaluation was based on visible grade criteria. The procedure-level recording improved objectivity, but quantitative mechanical damage detection, such as contact force sensing, image-based wound-area extraction, hyperspectral bruise detection or post-harvest shelf-life assessment, should be included in future work. Finally, the prototype has not yet been fully integrated with upstream tray separation and downstream automatic collection or packaging.

Future work will focus on multi-factor optimization using orthogonal or response-surface experiments, adaptive control of brush speed and belt speed according to crop morphology, force-feedback clamping to reduce stem indentation, multi-variety parameter libraries and continuous-line testing under production conditions. These improvements are expected to enhance robustness and support industrial application in hydroponic leafy-vegetable harvesting.

## 5 Conclusions

An orderly harvesting device integrating rotating root flicking, clamping conveying and differential steering was developed for hydroponic leafy vegetables. The conclusions are summarized from three aspects.

### 5.1 Mechanism innovation

The device combines non-cutting bristle root cleaning, compliant double-belt clamping conveying and continuous differential belt steering. The three modules are connected through lateral, height and twist-angle adjustment mechanisms, allowing root treatment, low-damage conveying and posture correction to be completed in one continuous process.

### 5.2 Experimental findings

The brush-speed comparison showed that 800 r/min provided a balanced rootflicking condition, with a root removal rate of 77.4% and a damage rate of 2.5%. The twist-angle comparison showed that 60^◦^ was the preferred clamping-conveying angle for both crops. Under this condition, spinach achieved a clamping-conveying success rate of 93.5%, a damage rate of 3.0% and a twist-angle MAE of 1.22^◦^, while Shanghai bok choy achieved 89.0%, 4.0% and 1.94^◦^, respectively. The differential steering comparison showed that an upper steering-belt speed of 325 mm/s was preferred. Under this condition, spinach achieved a steering success rate of 93.0%, a damage rate of 4.5% and an inclination-angle MAE of 1.15^◦^, while Shanghai bok choy achieved 94.5%, 3.5% and 0.98^◦^, respectively.

### 5.3 Practical value

The results demonstrate that the proposed device can complete coordinated root flicking, stable clamping conveying and posture steering for hydroponic leafy vegetables with low visible damage. The device provides a technical basis for continuous and orderly harvesting in controlled-environment leafyvegetable production, and the parameter-gradient results provide references for subsequent multi-factor optimization and multi-variety adaptation.

## Data Availability

The data presented in this study are available from the corresponding author upon reasonable request.

## References

[B1] Al-Kodmany K. (2018). The vertical farm: A review of developments and implications for the vertical city. Buildings 8, 24. doi: 10.3390/buildings8020024 30654563

[B2] Ampim P. A. Y. Obeng E. Olvera-Gonzalez E. (2022). Indoor vegetable production: An alternative approach to increasing cultivation. Plants 11, 2843. doi: 10.3390/plants11212843 36365296 PMC9657353

[B3] Barbosa G. L. Gadelha F. D. A. Kublik N. Proctor A. Reichelm L. Weissinger E. . (2015). Comparison of land, water, and energy requirements of lettuce grown using hydroponic vs. conventional agricultural methods. Int. J. Environ. Res. Public Health 12, 6879–6891. doi: 10.3390/ijerph120606879 26086708 PMC4483736

[B4] Baumont de Oliveira F. J. Ferson S. Dyer R. A. D. Thomas J. M. H. Myers P. D. Gray N. G. (2022). How high is high enough? assessing financial risk for vertical farms using imprecise probability. Sustainability 14, 5676. doi: 10.3390/su14095676 30654563

[B5] Beacham A. M. Vickers L. H. Monaghan J. M. (2019). Vertical farming: A summary of approaches to growing skywards. J. Hortic. Sci. Biotechnol. 94, 277–283. doi: 10.1080/14620316.2019.1574214 37339054

[B6] Benke K. Tomkins B. (2017). Future food-production systems: Vertical farming and controlledenvironment agriculture. Sustain. Sci. Pract. Policy 13, 13–26. doi: 10.1080/15487733.2017.1394054 37339054

[B7] Bunge A. C. Wood A. Halloran A. Gordon L. J. (2022). A systematic scoping review of the sustainability of vertical farming, plant-based alternatives, food delivery services and blockchain in food systems. Nat. Food 3, 933–941. doi: 10.1038/s43016-022-00622-8 37118205

[B8] Dutta M. Gupta D. Sahu S. Limkar S. Singh P. Mishra A. . (2023). Evaluation of growth responses of lettuce and energy efficiency of the substrate and smart hydroponics cropping system. Sensors 23, 1875. doi: 10.3390/s23041875 36850471 PMC9967833

[B9] Feng X. Xie C. Tong J. Guo S. Qi B. Gao Y. . (2025). Design and test of vertical axis rotating cutters for cutting corn roots and crown. Agriculture 15, 717. doi: 10.3390/agriculture15070717 30654563

[B10] Gao G. Li Z. Zhang Z. Lu X. Zhai S. Zhu Z. (2024). Optimal design of the response surface for the root cutting of a hydroponic lettuce harvesting tool. Proc. Inst. Mech. Eng. Part. B. J. Eng. Manuf. 238, 2152–2162. doi: 10.1177/09544054231222046

[B11] Guan X. Shi L. Ge H. Ding Y. Nie S. (2025). Development, design, and improvement of an intelligent harvesting system for aquatic vegetable Brasenia schreberi. Agronomy 15, 1451. doi: 10.3390/agronomy15061451 30654563

[B12] Gumisiriza M. S. Ndakidemi P. A. Mbega E. R. (2022). A simplified non-greenhouse hydroponic system for small-scale soilless urban vegetable farming. MethodsX 9, 101882. doi: 10.1016/j.mex.2022.101882 36311266 PMC9596717

[B13] Hosseinzadeh S. Verheust Y. Bonarrigo G. Van Hulle S. W. H. (2017). Closed hydroponic systems: Operational parameters, root exudates occurrence and related water treatment. Rev. Environ. Sci. Bio/Technol. 16, 59–79. doi: 10.1007/s11157-016-9418-6 30311153

[B14] Jin Y. Song Z. Zhang R. Zhang J. (2024a). Experiment and analysis of physical, mechanical, and viscoelastic properties of the roots and stalks of green leafy vegetables. PLoS One 19, e0305572. doi: 10.1371/journal.pone.0305572 38954711 PMC11218987

[B15] Jin Y. Wang J. Chen J. Song Z. Zhang R. Zhou R. (2024b). Design and experiment for flexible clamping and conveying device for green leafy vegetable orderly harvester. Agriculture 14, 967. doi: 10.3390/agriculture14060967 30654563

[B16] Khan H. A. Farooq U. Saleem S. R. Rehman U. Tahir M. N. Iqbal T. . (2024). Design and development of machine vision robotic arm for vegetable crops in hydroponics. Smart Agric. Technol. 9, 100628. doi: 10.1016/j.atech.2024.100628 38826717

[B17] Kozai T. Niu G. Takagaki M. (2019). Plant Factory: An Indoor Vertical Farming System for Efficient Quality Food Production. 2. Eds. Kozai T. Niu G. Takagaki M. (London, UK: Academic Press).

[B18] Li Y. Zhang J. Xie F. Yu Y. (2025). Design and experimentation of root-cutting device for head-forming vegetables. INMATEH Agric. Eng. 75, 831–840. doi: 10.35633/inmateh-75-71

[B19] Liu Y. Xia H. Feng J. Jiang L. Li L. Dong Z. . (2023). An enveloping, centering, and grabbing mechanism for harvesting hydroponic leafy vegetables cultivated in pipeline. Agronomy 13, 476. doi: 10.3390/agronomy13020476 30654563

[B20] Liu Y. Yao L. Yang Y. Wang S. Zhou J. (2025). Design and experiment of a clamping-conveying device for orderly harvesting of leafy vegetables. J. Agric. Mech. Res. 47, 155–164. doi: 10.13427/j.issn.1003-188X.2025.10.020

[B21] Lu D. Wang W. Li Y. Ou M. Ma J. Bao E. . (2025). Design and experiment of self-propelled high-stem Chrysanthemum coronarium orderly harvester. Agriculture 15, 1848. doi: 10.3390/agriculture15171848 30654563

[B22] Ma Y. Wang X. Zhang C. Wang H. Zhou H. Wang Y. (2026). Design and experiment of a posture adjustment and differential steering device for orderly harvesting of hydroponic leafy vegetables. Agronomy 16, 152. doi: 10.3390/agronomy16020152 30654563

[B23] Ma Y. Xu C. Zhang X. Li K. Fu L. Cui Y. (2020). “ An automatic harvesting system for hydroponic lettuce,” in Proceedings of the 2020 ASABE Annual International Virtual Meeting ( American Society of Agricultural and Biological Engineers, St. Joseph, MI, USA), 1.

[B24] Nikolov N. V. Atanasov A. Z. Evstatiev B. I. Vladut V. Biris S. (2023). Design of a smallscale hydroponic system for indoor farming of leafy vegetables. Agriculture 13, 1191. doi: 10.3390/agriculture13061191 30654563

[B25] Praveen K. Kumar Y. A. Shrivastava A. K. (2025). Development and evaluation of a battery powered harvester for sustainable leafy vegetable cultivation. Sci. Rep. 15, 33812. doi: 10.1038/s41598-025-03594-4 41028026 PMC12484774

[B26] Sabiev U. K. Khuzin I. R. Soyunov A. S. (2021). “ Technological process of interaction of the brush rod with the surface of the root crop,” in IOP Conference Series: Earth and Environmental Science, vol. 720. ( IOP Publishing, Bristol, UK), 012079. doi: 10.1088/1755-1315/720/1/012079

[B27] van Delden S. H. SharathKumar M. Butturini M. Graamans L. J. A. Heuvelink E. Kacira M. . (2021). Current status and future challenges in implementing and upscaling vertical farming systems. Nat. Food 2, 944–956. doi: 10.1038/s43016-021-00402-w 37118238

[B28] Wang W. Ma Y. Fu L. Cui Y. Majeed Y. (2021). Physical and mechanical properties of hydroponic lettuce for automatic harvesting. Inf. Process. Agric. 8, 550–559. doi: 10.1016/j.inpa.2020.11.005 38826717

[B29] Xia H. Li L. Deng C. Zhu S. Chen J. Yang T. . (2024). Finite element simulation parameter calibration and verification for stem cutting of hydroponic chinese kale. Agriculture 14, 422. doi: 10.3390/agriculture14030422 42495577

[B30] Xie L. Gao Y. Lin Z. He F. Duan W. Ye D. (2025). Design and test analysis of a rotary cutter device for root cutting of golden needle mushroom. Sci. Rep. 15, 37219. doi: 10.1038/s41598-025-21155-7 41136535 PMC12552425

[B31] Yu Y. Qi J. Li Y. Xie F. Song J. Bai Y. (2025). Design and experiment of key components for self-propelled harvester for chinese cabbage. Sci. Rep. 15, 17007. doi: 10.1038/s41598-025-02222-5 40379741 PMC12084294

[B32] Yu Z. Yang M. Hu Z. Gu F. Peng B. Zhang Y. . (2023). Kinematic analysis and process optimization of root-cutting systems in field harvesting of garlic based on computer simulation technology. Front. Plant Sci. 14, 1168900. doi: 10.3389/fpls.2023.1168900 37674735 PMC10477984

[B33] Zhang J. Wang J. Du D. Long S. Wang Y. Han C. . (2023). Optimization and validation of root-cutting device for chinese cabbage harvester based on discrete element method. Comput. Electron. Agric. 214, 108314. doi: 10.1016/j.compag.2023.108314 38826717

[B34] Zou L. Liu X. Li J. Niu Z. Song Y. Yuan J. (2019). Clamping-conveying device for an orderly spinach harvester based on rheological characteristics. Trans. Chin. Soc Agric. Mach. 50, 72–79. doi: 10.6041/j.issn.1000-1298.2019.10.008

[B35] Zou L. Yan D. Niu Z. Yuan J. Cheng H. Zheng H. (2023). Parametric analysis and numerical optimisation of spinach root vibration shovel cutting using discrete element method. Comput. Electron. Agric. 212, 108138. doi: 10.1016/j.compag.2023.108138 38826717

[B36] Zou L. Yuan J. Liu X. Li J. Zhang P. Niu Z. (2021). Burgers viscoelastic model-based variable stiffness design of compliant clamping mechanism for leafy greens harvesting. Biosyst. Eng. 208, 1–15. doi: 10.1016/j.biosystemseng.2021.05.007 38826717

